# Supplementary material for: Care needs of patients with chronic wounds for implementing a virtual care program: A qualitative study
Source: PLoS One. 2025 Dec 31;20(12):e0339895. doi: 10.1371/journal.pone.0339895 (PMC12755777; doi:10.1371/journal.pone.0339895)
Supplement: S1 File — (DOCX) [file pone.0339895.s001.docx]

**Annex 1. Semi-Structured Guide for Focus Group Discussions with Wound Therapists**

• Please describe your experiences working in a wound clinic and, if applicable, providing virtual or remote care to patients with chronic wounds.

• Please explain how much information patients with chronic wounds have about wounds and their management.

• How familiar were patients with different wound dressings, and how did patients perform in caring for their wound site?

• What care needs do patients with chronic wounds have to manage themselves and their wounds when providing virtual care?

• How do you value the presence of family members when providing virtual care to patients?

• What care activities might the patient or family members require assistance with to manage the patient’s wound remotely?

**Annex 2. Semi-Structured Interview Guide for Patients with Chronic Wounds**

• Please explain how your wound developed. And what did you do?

• Do you have any information about factors that affect wound healing?

• How has a chronic wound (diabetic foot ulcer, pressure ulcer, vascular ulcer, etc.) affected your activity and lifestyle?

• How do you care for your wound site?

• How familiar are you with the dressings used to dress your wound?

• What steps do you or your family members need to take to manage your wound at home?
