# Supplementary material for: Care needs of patients with chronic wounds for implementing a virtual care program: A qualitative study
Source: PLoS One. 2025 Dec 31;20(12):e0339895. doi: 10.1371/journal.pone.0339895 (PMC12755777; doi:10.1371/journal.pone.0339895)
Supplement: Attachment — Submitted filename: Comments from Reviewer.pdf [file pone.0339895.s002.pdf]

Comments to the Authors of the manuscript:

1. Abstract: Please add the number of FGDs and IDIs in the abstract on pages 31-32.
2. The Introduction is well written.
3. Line 100-102, please move ethical consideration to a separate paragraph at the end of the methodology section.
4. Settings and participants: Please add purposive sampling method was used for participant selection on line 109.
5. Page 6, line 115, is a repetition regarding informed consent. Please remove.
6. Page 7, I am not convinced how the FGDs were reported. Please follow the COREQ checklist to report your study. Please check. The original 2007 paper by Tong, Sainsbury, and Craig mentions that COREQ is a **32-item checklist for interviews and focus groups** in qualitative health research.
7. Please move the interview guide page 8, lines 149-158, to the annex.
8. Page 8, line 165 is a repetition, please remove.
9. Page 9, line 171, please report the mean duration of the interviews.
10. Page 9, move lines 172-179 to the annex.
